# Implementation of a standard outcome set in perinatal care: a qualitative analysis of barriers and facilitators from all stakeholder perspectives

**DOI:** 10.1186/s12913-021-06121-z

**Published:** 2021-02-02

**Authors:** Anne L. Depla, Neeltje M. Crombag, Arie Franx, Mireille N. Bekker

**Affiliations:** 1 Department of Obstetrics and Gynaecology, Wilhelmina Children’s Hospital, University Medical Centre Utrecht, KE.04.123.1, Lundlaan 6, 3584 EA Utrecht, The Netherlands; 2 Department of Development and Regeneration, KU Leuven University, Leuven, Belgium; 3 Department of Obstetrics and Gynaecology, Erasmus Medical Centre Rotterdam, Rotterdam, the Netherlands

**Keywords:** Health outcomes, Pregnancy, Obstetrics, Outcome measures, Implementation framework, Perinatal health, Patient-centred outcomes, Patient-reported

## Abstract

**Background:**

To improve their quality, healthcare systems are increasingly focused on value delivered to patients. For perinatal care, the International Consortium for Health Outcomes Measurement (ICHOM) proposed a patient-centred outcome set with both clinical and patient-reported measures for pregnancy and childbirth (PCB set). This study aimed to identify factors that affect the implementation of the PCB set at the pre-implementation stage, using the consolidated framework for implementation research (CFIR).

**Methods:**

In this qualitative study, we conducted semi-structured interviews amongst a purposive sample of key stakeholders within an obstetric care network (OCN): 1) patients, 2) perinatal care professionals involved in the full cycle of perinatal care, and 3) policy makers, including hospital managers, administrative staff and health care insurers. While the CFIR guided data capture and structuring, thematic analysis revealed overarching themes that best reflected the barriers and facilitators from different stakeholder perspectives. Within these overarching themes, the CFIR constructs were maintained.

**Results:**

Interviews were conducted with 6 patients, 16 professionals and 5 policy makers. Thematic analysis supported by the CFIR framework identified four main themes: the instrument and its implementation process, use in individual patient care, use in quality improvement, and the context of the OCN. Important barriers included professional workload, data reliability, and interprofessional and interorganizational collaboration. Potential facilitators were the PCB set’s direct value in individual care, interprofessional feedback and education, and aligning with existing systems. Prominent variations between stakeholder groups included the expected patient burden, the level of use, transparency of outcomes and the degree of integrated care.

**Conclusions:**

This study clarified critical factors that affect successful implementation of the PCB set in perinatal care. Practice recommendations, suggested at multiple levels, can enable structural patient-centred care improvement and may unite stakeholders towards integrated birth care.

**Supplementary Information:**

The online version contains supplementary material available at 10.1186/s12913-021-06121-z.

## Background

Worldwide, healthcare systems are shifting towards more value driven care [1]. After the era of evidence based medicine, healthcare stakeholders are aligning their goals in “learning health systems” that continuously measure and improve the value of care from the patients’ perspective [2–4]. In this journey, routine outcome collection from patients has become essential and empowers patients to take an active role in their care, e.g. via symptom detection and broader informed care decisions [3, 5, 6]. Therefore, patient-reported outcome measures (PROM) and experiences measures (PREM) – tools that assess patients’ perceived health status and their experience with received care – are progressively being used for clinical practice, research and quality improvement [7–9].

For perinatal care, numerous quality indicators are available, as pregnancy and childbirth are worldwide drivers of morbidity and costs, and large practice variation exists. Until now these indicators mainly focused on structure and process measures, such as prenatal care utilization or caesarean section rate, and to a lesser extent on clinical outcomes like postpartum haemorrhage. While important parameters of medical performance, these indicators do not directly reflect all outcomes that matter to pregnant women – for example urine incontinence or mother-child bonding. They also often lack an improvement incentive for clinicians [10].

The International Consortium for Health Outcomes measurement (ICHOM) developed, through international collaborations among patients, clinicians and researchers, a more complete outcome set for Pregnancy and Childbirth (PCB) [11]. This set consists of standardized clinical metrics, PROMs and PREMs, addressing outcomes that matter to pregnant women and their child [12]. With five measurement moments throughout pregnancy until 6 months postpartum, it considers quality of care from the patients’ perspective, regardless of barriers between different care professionals and organizations involved in perinatal care. Potential benefits of such standard outcome sets can emerge at several levels. In individual patient care, structural PROM collection has shown to significantly improve patient-provider communication, detection of unrecognized symptoms and even clinical health outcomes [13, 14]. At organization level, data on both clinical and patient-reported outcomes have been shown to support informed decision-making and empower providers to improve care [4]. Ultimately, international standardization of outcome measures enables benchmarking, reduces practice variation and creates learning health systems on the impacts that matter to patients.

Although the potential benefits of the PCB outcome set are recognized by key stakeholders in perinatal care, knowledge and instruments are lacking for its implementation in clinical practice, especially the collection and use of its PROMs and PREMs [15]. Some patient-reported measures of the PCB outcome set were recently collected in perinatal studies, but were used anonymously for quality improvement or research goals only [16, 17]. Other care settings in which common barriers and facilitators to implement PROMs have been identified have been limited to chronic or planned care – such as cancer care and surgery [18, 19]. These settings differ considerably from perinatal care, which affects a relatively healthy population at start of care, and within which multiple care organizations combine planned and acute care in a short time period. In most studies the challenges and success factors for PROM implementation have mainly been studied from the clinician perspective. Yet, patients and policy makers have been shown relevant stakeholders for the successful implementation of PROMs as well, in particular in network settings [18, 20, 21].

This qualitative study aims to identify impeding and enabling factors affecting the implementation of the PCB outcome set in perinatal care. In this pre-implementation analysis, we explored variations in stakeholder perspectives by interviewing care professionals, patients and policy makers. This will generate knowledge of the contributing factors and different incentives from each stakeholder perspective, facilitating the development of more effective implementation strategies.

## Methods

### Study design

For this pre-implementation analysis, a qualitative study was performed to explore barriers and enablers to implement the PCB outcome set in perinatal care, and to elaborate perspectives of key stakeholders. Semi-structured interviews were conducted to enable the interviewees to share their own perspectives and attitudes towards the topics of interest [22]. Data collection, analysis and interpretation were guided by the Consolidated Framework for Implementation Research (CFIR), a framework of standardized constructs developed by meta-analysis of theory-based models from several disciplines and proven to support the implementation process [20]. It comprises 39 constructs, organized across 5 major domains (Table 1). The framework is widely used in implementation research and applies to each phase of implementation [23]. Prior to implementation, it supports identification of multi-level factors that can affect future implementation [24].

**Table 1:** CFIR domains and constructs, with aligning study entities

Domain (aligning study entity)	Construct
Intervention Characteristics (of the PCB outcome set)	- Intervention Source - Evidence Strength and Quality - Relative Advantage - Adaptability - Trialability - Complexity - Design Quality - Cost
Inner Setting (OCN practices)	- Structural Characteristics - Networks and Communications - Culture - Implementation Climate - Readiness for Implementation
Outer Setting (Dutch perinatal care)	- Patient Needs and Resources - Cosmopolitanism - Peer Pressure - External Policy and Incentives
Characteristics of Individuals (OCN stakeholders)	- Knowledge and Beliefs about the Intervention - Self-efficacy - Individual Stage of Change - Individual Identification with Organization - Other Personal Attributes
Process (aspects of implementing, delivering and evaluating the PCB outcome set)	- Planning - Engaging - Executing - Reflecting and Evaluating

Legend: *PCB* Pregnancy and childbirth; *OCN* Obstetric care network

### Intervention background: the ICHOM Pregnancy & Childbirth standard set

The PCB outcome set was composed by ICHOM, which aims to develop standard outcome sets for each particular disease or condition from patients’ perspective. The PCB outcome set, developed through a Delphi procedure with international experts and patient involvement, consists of one third clinical outcomes and two thirds PROMs and PREMs [12]. The clinical metrics are collected 6 weeks postpartum; the patient-reported items are assessed with questionnaires at five moments proposed by ICHOM (2 during pregnancy and 3 postpartum; from 28 weeks of gestation until 6 weeks postpartum) [11]. The information could be used at several levels: at individual patient level as part of usual care, aggregated data to measure and improve care performance and externally for benchmarking, quality reporting or value-based payment.

### Setting

This study was carried out from May to August 2017 in the obstetric care network (OCN) around the Wilhelmina Children’s Hospital in Utrecht, the Netherlands. Dutch perinatal care is organized in a distinct two-tier system, providing primary care through midwives for low-risk pregnancies and secondary/tertiary care through obstetricians in the hospital for high-risk pregnancies. Primary care midwives act as gatekeeper to specialist care and have their own professional autonomy, responsibilities and financial arrangements. They collaborate with their secondary/tertiary referring partners in an Obstetric Care Network (OCN). Over the last decade, a more integrated obstetric care system (a collaboration of community care midwives and hospital employed obstetric professionals in one care pathway) has been advised by the Ministry of Health and is partly being realized within OCNs [25, 26]. The OCN in this study consists of a tertiary hospital, 6 community midwifery practices and multiple maternity care assistance organizations. In the setting of an OCN, all aspects relevant for implementation could be explored, as the instruments’ purpose is to address perinatal care performance over the whole pregnancy and postpartum period.

### Participants

**Fig. 1:**
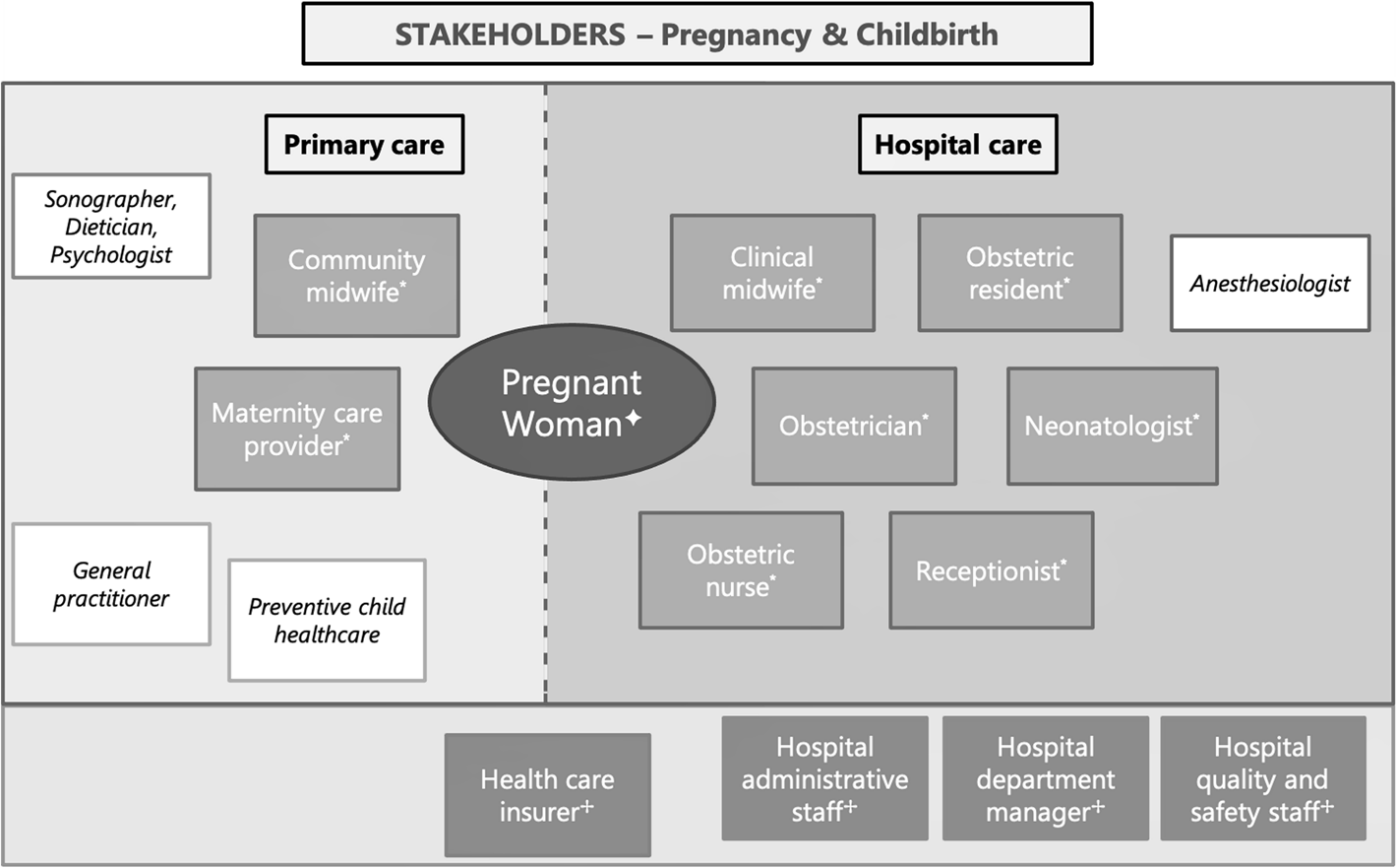
Stakeholder map. ‘filled’ boxes = key stakeholders (interviewed), ‘white’ boxes = stakeholders with minor involvement (not interviewed). Stakeholder groups: ✦Group 1 patients; *Group 2 care professionals; ✢Group 3 policy makers

All stakeholders involved with perinatal care in this OCN were systematically identified, according to a framework for stakeholder mapping in health research [27]. After defining stakeholder categories for perinatal care, both directly and indirectly involved stakeholders were mapped and feedback of expert informants was collected. During the interviews, this map was validated via snowballing sampling – i.e. new stakeholders arising from earlier interviews, until no relevant new stakeholders came up (Fig. 1). Key stakeholders comprised three main groups: patients, care professionals and policy makers. A purposive sample of patients was selected, including both pregnant and postpartum women, both nulliparous and multiparous, whether in primary or hospital care. Patients had to be 18 years old and able to speak Dutch. Professionals and policy makers were included based on their role in the OCN. Participants were included until saturation was reached. We anticipated six patients were needed and aimed to include two of each type of care professional or policy maker. Prior to each interview, participants received standardized background information about the study topic and verbal informed consent was obtained. None of the stakeholders received compensation for participation. Ethical approval for this study was granted by the University of Utrecht Ethics Committee.

### Data collection

A semi-structured topic list was composed that covered current quality improvement initiatives, levels of using the PCB outcome set, and determinants of change [see Additional file 1]. To guide complete data collection, this list was supported by an overview of the CFIR constructs and a selection of CFIR guide questions. For each CFIR domain, the aligning entity in this study is provided in Table 1. The interviews were conducted face-to-face at a location convenient to the interviewee and audio recorded after permission. All interviews were conducted by the first author, a researcher trained in interviewing and qualitative analysis. Every interview was transcribed verbatim using Amberscript software. After checking for accuracy by the researchers, the transcriptions were coded and stripped of personal identifying data.

### Analysis

Data analysis started directly after the first interview, using a combined deductive and inductive approach along the Qualitative Analysis Guide of Leuven (QUAGOL) [28]. This method, characterized by its iterative process and team approach, consists of two parts with five steps each: part one aims to create a conceptual understanding of the research data as a whole, part two is the actual coding process. In this study, the researchers read the transcripts and discussed first impressions, thoughts and initial codes. Then, the researchers identified themes in the transcripts, organized them along the CFIR framework and analysed differences between stakeholder groups. During this process, additional codes emerged to develop a thematic framework that better reflected the language and reflections of participants. Although the CFIR framework was identified as the a priori framework, our thematic analysis revealed four overarching themes best reflecting the topics our participants described. Within those overarching themes, we retained the CFIR constructs to maintain their in-depth value. The analysis process was executed with two authors (AD and NC) and supervised by a third author (MB). Constant movement between the various stages of the process was required as new data and themes emerged, resulting in interaction between each part of the analysis. The process was continued until saturation was reached. *NVIVO software (11.2.2)* facilitated data management, organization and analysis. Also, *Microsoft Excel (2010)* was used to organize constructs and compare stakeholder groups. Reporting followed the consolidated criteria for reporting qualitative research (COREQ) [29].

## Results

At 27 interviews, saturation was reached: 6 with patients, 16 with care professionals and 5 with policy makers involved in the OCN (Table 2). In this paper, interviewees are referred to as PT (patient), HCP or CCP (hospital-employed or community care professional) and PM (policy maker). Thematic analysis revealed four main themes: A) instrument and process factors, B) use in clinical practice, C) use aggregate outcomes for quality improvement, and D) context of the OCN. Although initially organizing along the CFIR framework, thematic analysis indicated significant overlap between the domains. As the complexity of the intervention and implementation context made it difficult to separate key findings by domain, the overarching themes found appeared most appropriate to describe our findings. The CFIR constructs identified within these themes are listed in Table 3. Each theme showed a variation in stakeholder perspectives: Table 4 provides an overview of the factors with prominent similarities *or* differences between stakeholder groups. A difference in perspective either meant a stakeholder group did not mention a barrier or facilitator, or they had another view (or focus).

**Table 2:** Number and function of individuals interviewed

Interview Subjects		Description
community care professionals (CCP)		
community midwife	2	provides perinatal care for low-risk pregnancy, delivery and postpartum care at home (also after discharge from the hospital)
maternity care provider	2	nurse that assists community midwife with at home deliveries and provides maternity care at home (also after discharge the form hospital)
hospital-employed care professionals (HCP)		
clinical midwife	2	all provide perinatal care to medium/high risk pregnancies and deliveries in the hospital
obstetrician	2	
obstetric resident	2	
obstetric nurse	2	
neonatologist	2	
receptionist	2	
policy makers (PM)		
hospital department manager	1	head of obstetric department
manager quality and safety	1	quality manager of the hospital
administrative staff	2	financial and clinical registration
healthcare insurer	1	largest regional insurer
patients (PT)	6	currently in perinatal care, equally representing: - pregnant and postpartum (within 6 weeks) - primiparous and multiparous - receiving hospital or community care, or both

**Table 3:** CFIR domains and constructs per theme; barriers and facilitators

Theme	Subthemes (facilitators and barriers)	CFIR elements identified (*domains*; constructs)
Instrument and process factors	Enabling: complete set; international consensus; instructions; effect proof; feedback professionals; patient engagement; combine registrations; interdisciplinary; leadership; IT-system	*Intervention characteristics:* intervention source, evidence strength, relative advantage, trialability, complexity, costs *Outer setting:* patient needs and resources, peer pressure *Inner setting:* implementation climate, readiness for implementation *Individual characteristics:* knowledge and beliefs, individual stage of change *Process:* planning, engaging
	Impeding: international consensus; effectivity; abstract; patient burden; resistance to change; professionals’ workload; lack of prioritizing; privacy; IT-system; costs	
Use in individual patient care	Enabling: patients’ benefits; time gain individual reaction; more unity	*Intervention characteristics:* relative advantage, complexity *Outer setting:* patient needs and resources *Inner setting:* implementation climate, readiness for implementation *Individual characteristics:* self-efficacy
	Impeding: PREM misinterpretation; professionals’ responsibility	
Use in quality improvement	Enabling: measures reflect goals; less fragmentation; motivation; improve quality; learn from benchmark; external policy	*Intervention characteristics:* relative advantage, complexity, cost *Outer setting:* patient needs and resources, external policy *Inner setting:* culture, implementation climate *Individual characteristics:* knowledge and beliefs about the intervention
	Impeding: data reliability; current QI; perceived influence; measures too general; transparency; scepticism PREMs	
Context of OCN	Enabling: local collaboration; trust; communication structures; more unity; integrated care	*Intervention characteristics:* relative advantage, complexity, cost *Inner setting:* structural characteristic, networks and communication, culture, implementation climate *Individual characteristics:* individual identification with organization
	Impeding: collaboration structure; financial incentives; interdisciplinary relations	

Legend: *OCN* Obstetric care network; *QI* Quality improvement; T5 = measurement moment at six months postpartum

**Table 4:**
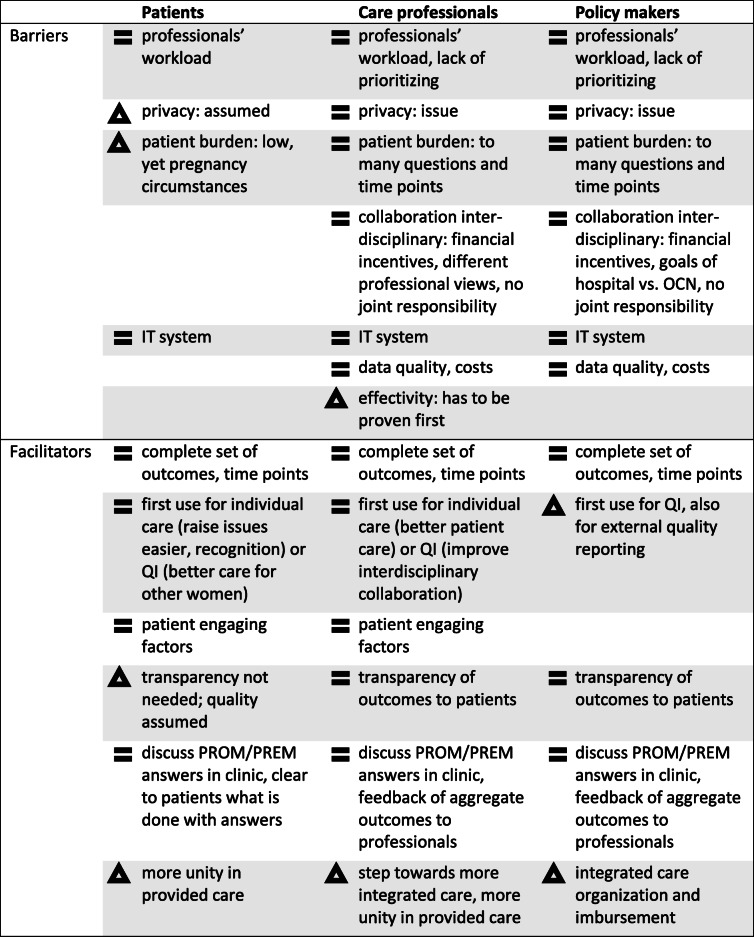
stakeholder perspectives

Legend: = similar perspective; = different perspective; *OCN* Obstetric care network; *QI *quality improvement.

### Theme A: instrument and process factors

All stakeholders appreciated, the PCB set combines clinical and patient-reported measures, covering most relevant aspects across the course of pregnancy. The set’s international, interdisciplinary development was considered to support uptake amongst care providers. Whilst policy makers were most keen about (inter)national uniformity, professionals noticed this can also hinder adaptation to a local context. To some, the instrument was still abstract and thought of as research, resulting in a passive attitude towards implementation. Professionals with basic understanding believed it can improve care and expressed willingness to start, emphasizing clear goals and instructions. Others demanded proof of efficacy first, for instance a pilot with quick feedback.

Thus, I do very much see the added value of this outcome set, not only to steer medical outcomes, but also experiences and… identify complaints women have by using it. HCP2

…I'm not going to try a new system... before it has been validated in a clinic. HCP3

For patients, both professionals and policy makers feared the questionnaire burden would be too high, especially for those with low socio-economic status. However, patients stated their willingness to complete five questionnaires of 5–15 min each. One patient anticipated circumstances around pregnancy, like postnatal depression, which might impede filling out the questionnaires. Similar strategies to engage patients were mentioned by professionals and patients: clear counselling about the purposes (both individual care and quality improvement), a personal approach, easy (digital) completion process and incorporation into usual care.

…one must be careful with the burden in time and intensity of questions you ask patients. CCP2

…I don’t think patients would complete four or five questionnaires. HCP3

I don't think it's all that many questions, I mean... you don't have to think about it for long. So that does not seem burdensome to me and a great good […] I would just make it obligatory. Yes, simply: fill in this list before your appointment, and if things are highlighted which we can discuss, we will do that. PT3

But if I feel like how I felt after my first child, I don't know whether I would be happy to do that (fill out a questionnaire). If I feel good, I am fine, I feel like it, I will do it. But back then, I really felt bad. PT1

At the same time, all participants raised concerns whether professionals have sufficient time to interpret and discuss individual answers, as well as to analyse data for quality improvement. Professionals’ workload and registration burden were underlined as already high, with a perceived lack of feedback and priorities in current improvement initiatives. Merging with existing systems and clinical processes was considered essential. All stakeholders identified an IT system with real-time data and guaranteed privacy as preconditions for implementation, but complex and costly to arrange in an OCN.

…because of the current workload you really see that... people don’t feel like it, people are tired… little leeway is left… people just keep their heads above water... HCP8

Well, as I said earlier, there are so many improvement projects going on: if this will be added again… those initiatives are all fantastic, but it seems a proliferation of… everything is called out like ‘this should be better, that should better, that can be better’ then I think ‘well, someone has to set priorities’… HCP2

### Theme B: use in individual patient care

All stakeholders recognized opportunities to detect symptoms earlier, to recognize individual issues and to adapt care accordingly. For patients and professionals, the standard questionnaires could make certain subjects – such as depression or incontinence, easier to raise. Provided before a visit, patient’s answers might enable professionals to gain time by focusing on the problems raised. Patients could become more aware of their health status and better prepared to pregnancy-related issues. Even more, patients valued comparing their health status to that of other women, feeling more recognized. With aggregated data on clinical and patient-reported measures, participants thought patients could make better-informed decisions. These benefits could empower women in their care process and increase their autonomy.

Some things you just don’t discuss so quickly… huh, that it’s still a bit of a taboo, to discuss or say or ask… depression in particular. PT5

I had that (depression) after my first child and... I was not heard, even though I indicated it. So the moment you report it here (questionnaire)… it’s easier for providers to recognize. PT1

…that I don’t have to deepen out that part of the anamnesis further… so it becomes easier to get to the core, indeed, of what it is about in those patients. HCP6

Regarding their PREM answers, patients worried about misinterpretation and wanted an opportunity to explain them. That way, they felt potential issues can be raised and dealt with earlier. Moreover, few patients proposed that all moments should include PREMs.

..*.*I would let that (PREMs) return particularly at the first and third moment as well. Because I noticed with the maternity care assistant at home: who actually asked every day like ‘are you satisfied, are there things I can do differently?’…that also gave space… if you are dissatisfied or if there are questions, to then still discuss that. PT3

…in perinatal care, and in other patient care as well: although you may not have done something optimally, if you find out with such a questionnaire and can reflect upon it and let a patient tell her story, she can still leave the hospital with a good feeling. So, I think you can use that, thus, on an individual level. HCP5

Providing individual patient’s answers to professionals and ensuring (re)action upon them was considered mandatory by all stakeholders. Yet, they also raised an obstacle in professional responsibility: it might be unclear which professional should interpret and act on answers, especially 6 months postpartum, when perinatal care has ended. However, all stakeholders expected increased collaboration and unity, as the questionnaires become a mutual responsibility of professionals across the network.

…because that does seem important to me, that you just also talk about it with a care professional, that it doesn’t linger. PT3

…the attunement between those... the midwifes have their image, and the gynaecologists have their image, and… one does not really prepare you for the other… expectation management can be improved... I think something like this (PCB set) can help with that. PT2

### Theme C: use aggregate outcomes for quality improvement

Compared to current indicators, professionals saw their efforts better reflected in the PCB set’s outcomes, increasing their motivation for registration and improvement initiatives. Those initiatives were expected to become less fragmented when approached from the patients’ perspective across the OCN, eventually leading to the most appropriate care. The purpose of quality improvement also increased patients’ motivation to complete questionnaires. For this use, obtaining reliable data was considered crucial, yet challenging due to selection bias and missing data, and requiring investments in IT and data management staff. To prevent increased registration and patient burden, several interviewees advocated dropping existing quality registrations. At the same time, some professionals would refuse to replace well-preforming intradisciplinary registrations, and policy makers noticed the external accountability of several performance measures.

…objective and subjective patient experience is a very important factor that we, I think, have taken aboard too little to date. HCP2

…actually, in particular also that group with a low SES (socio-economic status) or people with language problems, I would want to take along, […] and those are still the weaker groups that are very difficult to reach. CCP1

Nonetheless, professionals felt they have only a slight influence on (a part of) the PCB set’s domains and feared its outcomes are too general to lead care improvements, as they are assessed across provider organizations and lack process measures. However, most stakeholders believed insight into these outcomes would create awareness and identify areas for improvement. As an improvement strategy, professionals proposed joint education on specific domains, also creating more incentive for data collection. Additionally, they thought training in discussing taboo subjects would support them.

…what I’m a bit worried about… [is] that the outcomes are too general, too generic, to make them applicable for specific patient groups. HCP3

For further improvement, every stakeholder group valued that the instrument enables benchmarking to learn from other regions. Still, some professionals feared unfair data and increased competition between providers. Other professionals and policy makers advocated public transparency to create incentive for improvement. However, patients stated they wouldn’t choose their care provider based on these outcomes and, furthermore, worried their data would be shared with healthcare insurers. If used for external performance reporting, some in each stakeholder group mentioned scepticism about PREMs becoming equally as important as clinical outcomes.

…I think one should be careful with a kind of patient-snitch, so to speak, say marketing in healthcare… I’m not an advocate of that… and this (PCB set) can also facilitate that a bit. CCP2

…well, when you ask for advice it’s just: ask around, see which ones are near you, check the website. I think if you do a full comparative study of all possibilities and outcomes... you will go completely crazy. PT3

### Theme D: context of the obstetric collaborative network

All stakeholder groups emphasized, because of the joint responsibility for pregnancy and childbirth as a whole, implementation across the OCN. All the same, the OCN was considered a complex context, as multiple organizations collaborate with no joint juridical entity. Consequently, professionals and policy makers noticed issues with data ownership, allocation of costs, patient flow in and out of the network and various medical record systems. Furthermore, they pointed out that different incentives exist between OCN and hospital, whilst community midwives are autonomous as well. When joint financial rewards are lacking, it was argued that joint improvement cycles remain restricted.

…as long as community practices maintain their own financial autonomy, you always have... uh, other interests at play. Not only your quality interest, but also financial interest... So, introducing the PCB set will improve quality to some extent, but on very relevant points... other interests are greater… HCP3

Whereas trust was identified key for joint outcome improvement, professionals perceived a barrier in interdisciplinary relationships within the OCN. Despite a decade of collaboration, professionals’ views on pregnancy and childbirth still differ, resulting in different care policies and lack of trust. Most professionals felt partly related to the OCN, depending on who they worked with in daily practice, and still identified closest with their organization or professional group. Policy makers, most hospital employed, perceived their few OCN tasks as inconvenient or complex. They recognized interprofessional collaboration barriers but lacked incentive or tools for change. At the same time, some collaboration was seen as performing very well – bringing local collaboration and more interdisciplinary equality and trust – for example joint audits and knowing each other personally and professionally, and the multiple communication systems established across the OCN to reach each professional group.

…an enormous translation has been made in uniformity of interdisciplinary protocols [...] there is still some improvement possible, because in the end the clinical point of view always prevails in my opinion... and I don't always think that is justified... CCP2

…that (integrated care) is very much stimulated by the government, but it certainly felt like a kind of forced collaboration… especially among the gynaecologists and midwives, who struggled very much with ‘how you do that’? And that is often on financial grounds, I noticed. HCP9

I think we all want to, but also don’t always say so… I guess many things are thought, but not everything is spoken out. CCP3

…the collaboration with the hospital, there is always something above it: who has the power here? PM1

Despite structural and cultural barriers, all stakeholders acknowledged the potential of the PCB set to strengthen interdisciplinary collaboration within the OCN by shared responsibility for outcomes. Patients expected it might improve interprofessional collaboration and continuity in care policy and advice. A health care insurer suggested eventually merging to one organization, to overcome structural and financial barriers and make future value-based payments possible. This, it was argued, would provide improvement incentives, truly arranged from the patients’ perspective. Though professionals considered this too soon, they saw the PCB set as a positive step towards more integrated care.

I think you want the best outcomes together… in that way you will also go to an integrated organization faster, because you really have to do it together. CCP3

…for that (bundled payment) the OCN actually has to be an organization instead of a collaboration… and because you also have a joint, eh, contract then… they also feel jointly responsible. PM5

## Discussion

**Fig. 2:**
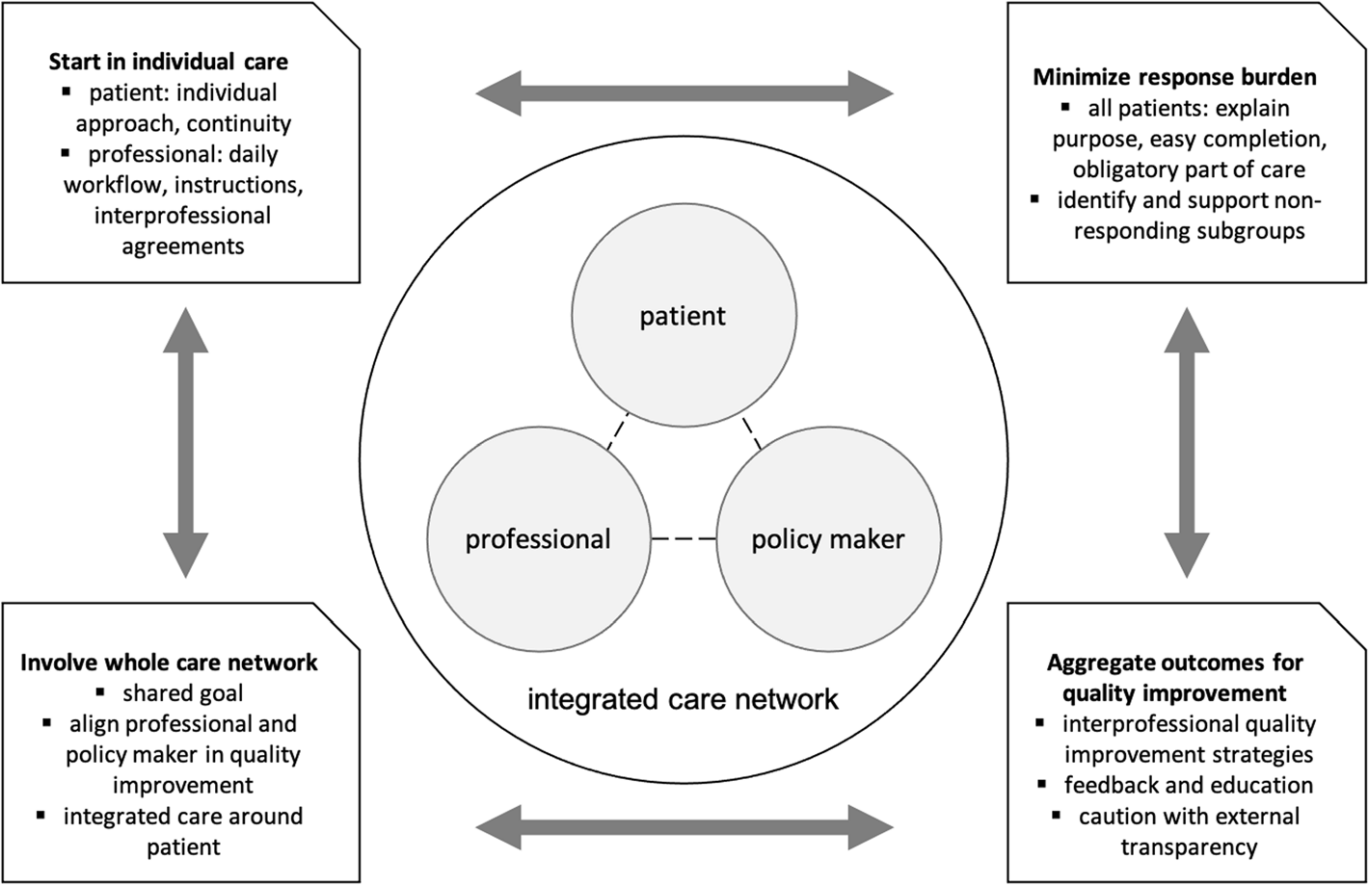
Recommendations for practice

This pre-implementation analysis systematically explored factors affecting successful implementation of the use of the PCB outcome set in perinatal care. Supported by the CFIR framework, a complete overview of interrelated constructs was identified across four main themes: instrument and process, use in clinical practice, use for quality improvement and the context of the OCN. Important barriers included local adaptability, feared patient burden, privacy, professionals’ workload and responsibilities, limited influence on outcomes, data reliability and transparency, financial incentives, collaboration structure and cultural differences. At the same time, it offered the completeness and relevance of the PCB sets’ outcomes, direct value to individual care, possibilities for professional education and feedback, patient engagement, integration into the clinical workflow, IT-systems and interprofessional shared goals. Here, we further elaborate stakeholders’ perspectives and factors unique for this setting, and can make recommendations based on our findings (Fig. 2)*.*

Despite professionals and policy makers raising patient burden as a substantial barrier, patients considered the questionnaires’ length and frequency appropriate. Studied in other settings, patients also seem to perceive the response burden of completing many PROMs as minimal, especially when their answers are used to guide clinical care [30, 31]. Still, non-response and partial completion often hinder the adoption and sustainability of PROMs [18]. In recent studies, perceived response burden and completion rates have been shown to correlate with health status, cognitive function, treatment factors and demographic characteristics [30, 32]. Hence, rather than the length or subjects of a questionnaire, patient characteristics and circumstances were predictive for PROM completion. In perinatal care, these factors could include pregnancy related illnesses, low literacy and socio-demographic background [33]. With future implementation, efforts should be made to identify and understand non-responding patient groups or pregnancy circumstances, in order to tailor strategies to support them – for example, with in-clinic assistance, questionnaire translations or an interview setting (Fig. 2). For all patients, response burden can be minimized by discussing outcomes individually to let women feel their story matters.

Across all stakeholder groups, using individual answers to guide patient care was believed to engage both patient and professional. This way, having PROMs’ value directly visible in clinical practice, was also considered an important facilitator in previous implementation research [18, 34]. However, to date individual use of PREMs has been limited, because of the fear to yield socially desirable answers as a result of the dependency relation between patient and professional [15, 35]. Interestingly, our patients emphasized the opportunity to explain PREM answers face-to-face and, furthermore, felt supported to raise negative experiences if they become part of clinical routine. These women might have become accustomed to daily individual experience evaluations with maternity care assistance. Therefore, discussion of PREMs individually might be optional, providing women a choice whether to show their answers to their provider or only use them anonymously for quality improvement. At the same time, this use in clinic requires clear instructions and easy data access for professionals, embedded in daily workflow (Fig. 2). Furthermore, care pathways and actions following the outcomes should be agreed on interprofessionally to ensure continuity of care and follow-up of patients’ answers throughout the network, for instance with a principal care provider.

When using aggregate outcomes for quality improvement, public transparency was debated by our participants and could have bidirectional impact on implementation. Whilst some professionals feared competition and fragmentation, public reporting was seen by others as stimulating improvement at the organization level, in line with a review on how performance data can improve care [36]. According to some, however, this information would not be used by patients to choose providers, as they mainly rely on relatives’ experiences, something affirmed by patients both in our study and in other papers [37]. Like patients in other settings, women did value aggregate outcomes to compare themselves to others and make treatment decisions [38]. Thus, the value of public reporting is questionable for choosing a provider, whereas its effect on quality of care seems bidirectional. Transparency can create tension for improvement on a managerial level, as well as unintended competition and fragmentation of care networks. In a slowly growing interprofessional collaboration, public reporting should therefore not be prioritized, as it could impede continuity and quality of care. Aside from this, the value of aggregate outcomes was recognized as a way to gain insight and awareness of patient-reported outcomes and to identify multidisciplinary opportunities for improvement. This stakeholder motivation advocates starting with regular feedback to all disciplines involved, with interprofessional education around domains of the PCB set (Fig. 2). Such a strategy would be supported by a review of facilitators in quality improvement using outcome indicators, although to date, PROMs have been rarely incorporated in structural improvement strategies [39].

While stakeholders all favoured implementing the PCB set across the OCN, important structural and cultural organization barriers arose within this complex context, crossing the boundaries of public health, community care and hospital care. Notably, these organizational aspects have been given little attention in other studies on PROM implementation, mostly conducted within organizations [18]. In integrated care networks, similar factors have been shown to affect interprofessional and interorganizational collaboration for a long time, not only in the Netherlands but also in perinatal care systems elsewhere [40–42]. Barriers like financial autonomy and limited trust could be addressed with interdisciplinary education or efforts to increase mutual acquaintance, yet are unlikely to be solved completely with any implementation strategy in the near future [41, 42]. Nonetheless, with the PCB set providing a more patient-centred approach, barriers could be reduced in future as shared responsibility for outcomes provides opportunities to unite towards integrated care [42]. Therefore, involving the whole integrated care network needs to be the focus, aligning professional and managerial incentives around the patient’s perspective (Fig. 2). Though policy makers seemed to adopt interprofessional attitudes, it could be their role in particular to bridge differences and provide leadership from the OCN. Fragmentation could decrease as the implementation of the PCB set enables measurement of a joint goal, supporting the journey towards integrated value driven care.

### Strengths and limitations

Although women were randomly selected from a varied population and included up to saturation, caution is always needed regarding the generalizability of qualitative methods. Patients should actively participate in further implementation evaluation. To obtain a complete view on patients’ needs and beliefs, purposive sampling of patients with both favourable and unfavourable PROM or PREM results would be of added value. Unfortunately, this was not possible in our pre-implementation study as the questionnaires had not been filled out by patients yet. While combined methods may have added to the generalizability, the semi-structured interviews provided us with an in-depth understanding of the various perspectives [22]. At this stage of implementation, it was most valuable to gain deeper understanding of participants’ motives and beliefs, rather than quantitative results.

A strength of this study was that stakeholders were identified systematically, reflecting the views of different professionals and policy makers as well as patients. Including patients was crucial, since in successful implementation, they have been shown to be equally important stakeholders. Aligning the incentives of professionals and policy makers has been reported crucial but is also often lacking [43]. Furthermore, the CFIR framework supported complete assessment of what is needed to implement changes in the context of perinatal care. Thereby, we extended the frameworks’ use to an integrated network setting, including care providers collaborating over a whole cycle of care; this is momentous in the current transformation to value driven healthcare [21].

## Conclusions

Before implementing the PCB outcome set, this qualitative study explored contributing factors and different incentives from each stakeholder perspective. This allows for both addressing barriers early and tailoring implementation strategies to the unique context of perinatal care. As our findings indicate, implementing the PCB set can be valuable to all stakeholders in perinatal care, providing an opportunity to improve individual patient care and to unite providers towards more integrated care around their patient. Implementation could start in clinical practice and involve the whole care network in quality improvement strategies. Future research should monitor this implementation process, inquiring into both interprofessional collaboration and the effects on patient outcomes.

## Supplementary Information

**Additional file 1: **Topic list semi-structured interviews.

## Data Availability

The datasets used and analysed during the current study are available from the corresponding author on reasonable request.

## Abbreviations

PROM: Patient-reported outcome measure
PREM: Patient-reported experience measure
ICHOM: International consortium for health outcomes measurement
PCB: Pregnancy and childbirth
CFIR: Consolidated framework for implementation research
OCN: Obstetric care network
QUAGOL: Qualitative analysis guide of leuven
COREQ: Consolidated criteria for reporting qualitative research
