# Supplementary material for: Implementation of a standard outcome set in perinatal care: a qualitative analysis of barriers and facilitators from all stakeholder perspectives
Source: BMC Health Serv Res. 2021 Feb 2;21:113. doi: 10.1186/s12913-021-06121-z (PMC7852077; doi:10.1186/s12913-021-06121-z)
Supplement: Supplementary file 1 — Additional file 1: Topic list semi-structured interviews. [file 12913_2021_6121_MOESM1_ESM.docx]

**Additional file 1 – Topic list semi-structured interviews**

*Prior to the interview, participants received standardized background information about the PCB set, its development and purpose.*

**Part 1:** Knowledge and current situation

- Current quality improvement policies (registrations, audits, protocols)
- Knowledge of the PCB set (purpose, content, source)

*Main question:* what do you know about quality improvement in perinatal care?

*Main goal:* insight in understanding of PCB set and quality improvement, insight in current situation

**Part 2:** Levels of using the PCB set outcomes

- Added value of the PCB set at:
  - Patient level
  - Organization or OCN-level
  - Benchmarking of several institutions or regions
- Level of preference of stakeholder

*Main question:* How do you think the PCB set can contribute to better quality of birth care?

*Main goal:* how does ICHOM fit in current quality improvement; preference for type of use; intrinsic motivation, personal goals, incentives for change

**Part 3:** Determinants of change

- Barriers
- Facilitators
- Other stakeholders involved
- Responsibilities and role of stakeholder
- Incentives stakeholder
- Patient (interest, burden)
- Overall opinion on the proposed intervention

*Main questions:*

- What do you think is necessary for a successful implementation of a quality cycle based on the PCB set?

- What bottlenecks do you see for this implementation? Which factors can promote implementation?

- How do you feel about the intervention?

*Main goal:* bottlenecks and facilitators for the intervention, new stakeholders, drivers and role of stakeholder, Overall opinion on the proposed intervention
